# Preoperative PET/CT ^18^F-FDG Standardized Uptake by Lymph Nodes as a Significant Prognostic Factor in Patients with Colorectal Cancer

**DOI:** 10.1155/2018/5802109

**Published:** 2018-11-01

**Authors:** Ruohua Chen, Yining Wang, Xiang Zhou, Gang Huang, Jianjun Liu

**Affiliations:** ^1^Department of Nuclear Medicine, Ren Ji Hospital, School of Medicine, Shanghai Jiao Tong University, Shanghai, China; ^2^Shanghai Key Laboratory for Molecular Imaging, Shanghai University of Medicine and Health Sciences, Shanghai, China

## Abstract

**Purpose:**

We evaluated the prognostic value of preoperative ^18^F-FDG uptake by suspected lymph nodes (LNs) using ^18^F-FDG PET/CT in colorectal cancer patients.

**Methods:**

Patients with CRC underwent ^18^F-FDG PET/CT before radical surgery. We used Cox proportional hazards regression to examine the relationship between recurrence and the ^18^F-FDG maximum standardized uptake value (SUVmax) in the suspected LNs (SUV_LN_) on ^18^F-FDG PET/CT.

**Results:**

Clinical data, treatment modalities, and results from 90 CR C patients were reviewed. The median follow-up was 19 months (range 3 to 72 months). Receiver operating characteristic analysis identified SUV_LN_ 1.15 was the optimal cut-off value for predicting recurrence. SUV_LN_ correlated with tumour size (*P*=0.045), lymph node metastasis (*P*=0.03), and recurrence (*P* < 0.0001). Univariate analysis showed significant associations between recurrence and SUV_LN_ (*P*=0.017), and tumour grade (*P*=0.013). Multivariate analysis identified SUV_LN_ (*P* < 0.0001), and tumour grade (*P*=0.005) as independent risk factors for recurrence. Patients with SUV_LN_ ≤ 1.15 and SUV_LN_ > 0.15 differed significantly in terms of recurrence (*P* < 0.0001).

**Conclusion:**

Preoperative SUV_LN_ measured by ^18^F-FDG PET/CT was significantly associated with recurrence and had significant prognostic value for recurrence-free survival in patients with colorectal cancer.

## 1. Introduction

Colorectal cancer (CRC) is one of the most common malignant tumours worldwide [1, 2]. Surgery and chemotherapy are the main two strategies for its treatment. However, treatment outcomes for CRC remain unsatisfactory, because of recurrence and metastasis, particularly in patients with advanced CRC [3–6]. The TNM classification has been widely used to estimate prognosis and to decide on treatment of malignant tumours [7]. Lymph node (LN) metastasis is one of the most important prognostic factors in CRC because the survival rate among CRC patients with lymph node metastasis was significantly lower the rate among those without lymph node metastasis [8–11]. Traditional imaging methods play an important role in detecting lymph node metastases of malignant tumours [12, 13]. However, these methods only reflect the size, density, and morphology of the lymph nodes; the biologic activity and aggressiveness of lymph nodes cannot be determined by traditional imaging methods. Thus, alternative imaging methods that better reflect the biologic behaviour of lymph nodes in CRC are of great importance.

Fluorine 18 (^18^F) fluorodeoxyglucose (FDG)–combined positron emission tomography (PET) and computed tomography (CT) (PET/CT) is based on the abnormally high rate of glucose metabolism found in cancer cells. It is widely used in diagnostic imaging of many malignant tumours [14–16]. Although previous studies showed that ^18^F-FDG PET had relatively low sensitivity in the assessment of nodal status in early-stage CRC, it showed better performance than conventional imaging methods, including CT [17, 18]. ^18^F-FDG PET/CT was recommended as a routine procedure in the management of CRC. However, most studies have focused on the relationship between the metabolic activity of the primary tumour and prognosis in CRC [19–21]. The metabolic activity of lymph nodes, measured as ^18^F-FDG uptake, possibly better reflecting the biologic behaviour or aggressiveness in CRC, has been rarely evaluated. We hypothesized that the metabolic activity of lymph nodes in patients with CRC may reflect the biologic behaviour or aggressiveness of the primary tumour and may have prognostic importance.

We investigated the relationship between ^18^F-FDG uptake by lymph nodes and clinicopathological characteristics in patients with CRC, and evaluated the significance of ^18^F-FDG uptake by lymph nodes for predicting recurrence in CRC patients.

## 2. Materials and Methods

### 2.1. Study Population

We included 90 patients (56 men and 34 women; age range, 43–87 y) with colorectal cancer. All had undergone ^18^F-FDG PET/CT before radical resection between 2011 and 2017. Patients were included when they met the following criteria: they had been treated by radical resection of colorectal cancer with lymphadenectomy; the diagnosis of colorectal cancer had been confirmed by histopathologic examination; complete case records, including data on age, sex, tumour location, tumour size, T-stage, lymph node metastasis, lymphovascular invasion, tumour grade, and adjuvant treatment, were available. The study was approved by the institutional review board of the Shanghai Jiaotong University–affiliated Ren Ji Hospital and was in accordance with the 2013 revision of the Declaration of Helsinki. Informed consent was waived in this study.

### 2.2. ^18^F-FDG PET/CT

^18^F-FDG PET/CT was performed using a whole-body scanner (Biograph mCT; Siemens Medical Systems). All patients received an intravenous 3.7 MBq/kg injection of ^18^F-FDG after having fasted for at least 6 h and rested for 1 h. The mean uptake time was 50 ± 6 min. Blood glucose levels were measured and were found to be less than 140 mg/dL at the time ^18^F-FDG was administered. The CT component of the scan was performed without contrast administration at 120 kV, 140 mA, and a section thickness of 5.0 mm to match the thickness of the PET images. PET image datasets were reconstructed iteratively, with the CT data applied for attenuation correction.

### 2.3. Image Analysis

For quantitative analysis, irregular regions of interest were placed over the most intense area of ^18^F-FDG uptake. SUVmax was calculated as (maximum pixel value with the decay-corrected region-of-interest activity (MBq/mL))/(injected dose (MBq)/body weight (kg)). SUV_LN_ was defined as the maximum standardized uptake value of suspected lymph nodes. As for the SUV_LN_, a single lymph node with the most avid FDG uptake was chosen for analysis and blood pool activity was used as the representative value of SUV_LN,_ if there were no visible lymph nodes on CT scan and there was no avid ^18^F-FDG uptake on PET [22]. The PET/CT images were evaluated by two experienced nuclear medicine physicians.

### 2.4. Lymph Node Dissection and Histologic Evaluation

All patients were treated with radical surgery and lymphadenectomy, according to tumour location. Primary tumour and each lymph node were sliced and stained with haematoxylin and eosin and examined microscopically by a pathologist. The numbers of lymph nodes retrieved in each area and the presence or absence of metastases were recorded.

### 2.5. Clinical Endpoints and Follow-Up

After surgical resection, all patients underwent clinical follow-up that included diagnostic imaging methods and blood tests after surgical resection. During follow-up, clinical assessment, including serum CEA, CA199, and CA724 levels, was performed every 2–3 months. Enteroscopy, contrast-enhanced CT, or MRI scans were performed every 6–8 months. Median follow-up was 19 months (range, 3–72 months). In addition, ^18^F-FDG PET/CT was performed if the clinical assessment or studies performed during follow-up showed an abnormal finding. Recurrent tumour and distant metastasis were diagnosed based on either a positive biopsy or unequivocal clinical or radiographic evidence of progression. The time to recurrence was defined as the time from the date of surgery to the date of recurrence.

### 2.6. Statistical Analysis

The relationship between the clinicopathological characteristics and recurrence were analysed by the *χ*^2^ test, the unpaired two-tailed test and the Mann–Whitney *U* test, where applicable. The Kaplan–Meier method with the log-rank test was used to explore the relationship between SUV_LN_ and recurrence. The Cox proportional hazard model was used to evaluate prognostic variables. Receiver operating characteristic (ROC) curve analysis was performed to determine the cut-off values for predicting recurrence. The receiver operating characteristic curve was used to assess the optimal threshold of SUV_LN_ with which to predict recurrence. Pearson correlation coefficient was used to measure the correlations between SUV_LN_ and clinicopathological characteristics. The data are represented as mean ± standard deviation. *P* < 0.05 was indicated significant difference. All statistical analyses were performed with SPSS software (SPSS, version 13.0; SPSS, Chicago, Ill).

## 3. Results

### 3.1. Patient Characteristics

The clinicopathological characteristics of the 90 enrolled patients are shown in Table 1. The median duration of follow-up was 19 months (range, 3–72). There were 44 (48.9%) patients with colon disease and 46 (51.1%) patients with rectal disease. Seventy-one (78.9%) were well- or moderately-differentiated and 19 (21.1%) were poorly-differentiated. The median tumour size was 4.67 cm (range, 1.2–12.0). There were 27 patients (30%) with lymph node metastasis and 12 patients (13.3%) with lymphovascular invasion. Nineteen patients (21.1%) suffered recurrence.

Among the 19 patients with recurrence, 17 (89.5%) experienced recurrence within the first two years. The most frequent site of recurrence was distant metastasis (*n*=9, 47.4%), followed by locoregional recurrence (*n*=6, 31.6%), and peritoneal recurrence (*n*=4, 21.0%). The most common site of distant metastasis was liver (*n*=4), followed by the lung (*n*=3), distant lymph node (*n*=1), and bone (*n*=1).

### 3.2. Differences between Nonrecurrent and Recurrent Patients

Table 2 depicts the patient characteristics and ^18^F-FDG PET/CT imaging grouped by CRC patients with or without recurrence. No significant differences between these groups were found in terms of age, sex, tumour location, tumour size, lymph node metastasis, lymphovascular invasion, adjuvant treatment, or SUV_Tumour_. However, a significant difference in SUV_LN_, T-stage, and tumour grade was found between these groups.

Table 3 summarizes the relationship between SUV_LN_ and site of recurrence in 19 patients with recurrence. In the 19 patients with recurrence, the Kruskal–Wallis test showed that there were no significant differences in SUV_LN_ between nine patients with distant metastasis (median, 3.42; range, 0.7–10.2), four patients with peritoneal recurrence (median, 3.0; range, 2.0–4.0), and six patients with locoregional recurrence (median, 3.59; range, 0.7–15.2; *P*=0.841).

### 3.3. Measurement of SUV_LN_ Cut-Off Value

**Figure 1:**
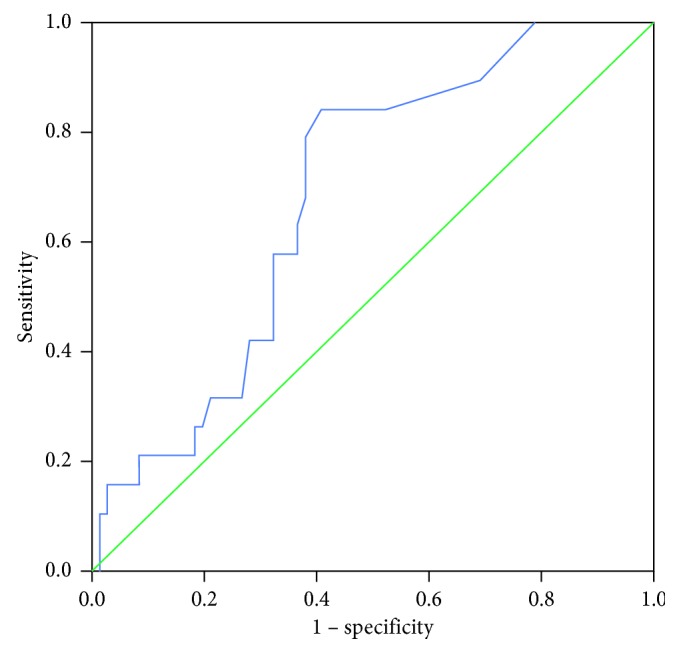
ROC curve analysis of recurrence prediction according to the ^18^F-FDG uptake of lymph node in 90 patients with CRC. The area under the curve was 0.683 (95%CI 0.562–0.803, *P*=0.015), and 1.15 was determined as the best SUV_LN_ cut-off value for predicting recurrence. With an SUV_LN_ of 1.15 as the threshold, sensitivity and specificity in the prediction of recurrence were 84.2% and 59.2%, respectively.

ROC analysis identified a cut-off value of 1.15 as significant for SUVLN (area under the curve 0.683; *P*=0.015; 95% CI 0.562–0.803; Figure 1). The sensitivity and specificity at this value were 84.2% and 59.2%, respectively. Based on the ROC curve analysis, the patients could be divided into two groups: SUV_LN_ ≤ 1.15 vs. SUV_LN_ > 1.15.

### 3.4. Prediction of Recurrence

**Figure 2:**
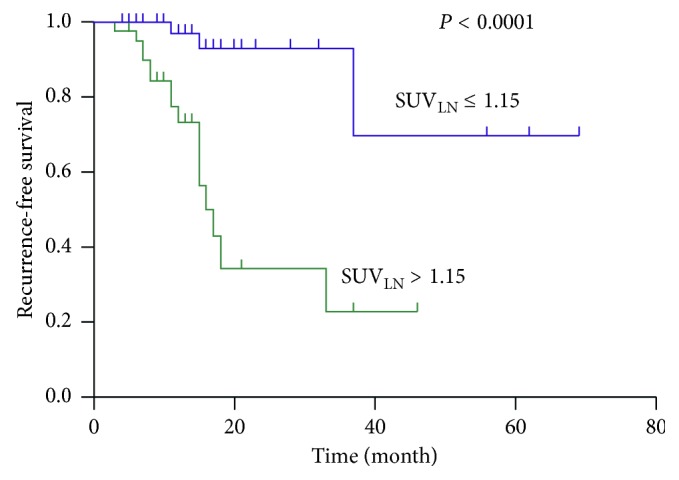
The Kaplan–Meier survival graph of the recurrence-free survival of patients with CRC stratified according to SUV_LN_. There as a statistically significant difference in recurrence-free survival between patients with SUV_LN_ > 1.15 (green line) and SUV_LN_ ≤ 1.15 (blue line) (*P* < 0.001, log-rank test).

Table 4 summarizes the results of the Cox proportional hazard model of prognostic factors for recurrence-free survival. Optimal cut-off values were 4.25 cm for tumour size and 10.65 for SUV_Tumour_, as determined by receiver operating characteristic curve analysis. Among clinicopathological characteristics and ^18^F-FDG PET/CT parameters, SUV_LN_ (*P*=0.017), and tumour grade (*P*=0.013) were risk factors for recurrence at univariate regression analysis. T-stage showed marginal significance (*P*=0.054). Multivariate analysis showed that SUV_LN_ (*P* < 0.0001) and tumour grade (*P*=0.005) were the independent risk factors for recurrence. The patient group categorized by SUV_LN_ showed a significant difference in PFS (log-rank test, *P* < 0.0001) as shown in the Kaplan–Meier survival curves (Figure 2).

### 3.5. Correlations between SUV_LN_ and Clinicopathological Characteristics

Table 5 depicts the correlation between SUV_LN_ and clinicopathological characteristics in patients with CRC. There were significant correlations between SUV_LN_ and tumour size (*P*=0.045), lymph node metastasis (*P*=0.03), and recurrence (*P* < 0.0001).

## 4. Discussion

The purpose of our study was to determine if preoperative metabolic activity in the lymph node as measured in terms of SUVmax on ^18^F-FDG PET/CT had prognostic significance in patients with CRC. The preoperative evaluation of the metabolic activity of suspected LN by ^18^F-FDG PET/CT was found to be a highly accurate prognostic tool for predicting recurrence in colorectal cancer. To the best of our knowledge, this study was the first to investigate the prognostic value of preoperative SUV_LN_ for recurrence risk in colorectal cancer treated with radical surgery.

Several studies have evaluated ^18^F-FDG PET in prognosis of patients with CRC. ^18^F-FDG uptake of tumour lesions was a significant prognostic factor for CRC patients who underwent curative surgical resection [19, 23]. In the present study, SUV_Tumour_ was not associated with recurrence in patients with CRC. This may be due to the small number of enrolled patients. The principle finding was that preoperative SUV_LN_ measured on ^18^F-FDG PET/CT was the most significant factor for predicting recurrence in colorectal cancer. Though the TNM classification has been widely used in the clinical setting to estimate prognosis, and previous studies have demonstrated that lymph node metastasis was a well-known risk factor for disease-free survival and overall survival in patients with CRC [7–10], contrary to these prognostic factors, ^18^F-FDG PET was advantageous in providing prognostic information even before surgery. Although several studies have evaluated the relationship between the recurrence and the SUV of lymph node in patients with CRC pathologically confirmed node-positive disease [24, 25], our study showed that preoperative SUV_LN_ measured on ^18^F-FDG PET/CT could predict the recurrence and it is not necessary to consider whether lymph node is positive.

In our study, the Pearson correlation coefficient provided strong evidence that SUV_LN_ was positively associated with lymph node metastasis, tumour size and recurrence. These results suggest that the metabolic activity of lymph node can be a useful functional marker of tumour aggressiveness. In this respect, the preoperative metabolic activity of suspected lymph node in patients with CRC, as measured by ^18^F-FDG PET/CT, may become a novel and promising functional biomarker for predicting recurrence before surgery.

The size, density, and morphology of the lymph nodes in malignant patients with early-stage may not change significantly, and thus, in such patients CT or MRI may prove less accurate in detecting metastatic lymph nodes. Therefore, care is necessary in interpreting ^18^F-FDG PET/CT results, especially when the lymph node size is small and the lymph node morphology is normal. False-negative findings are mainly due to the limited spatial resolution of ^18^F-FDG PET/CT. Therefore, preoperative metabolic activity of suspected lymph nodes in CRC patients measured by ^18^F-FDG PET/CT scan may be a novel biomarker for predicting recurrence. When the SUV_LN_ in CRC patients increased, the recurrence risk increased significantly. This result emphasized the importance of the metabolic activity of lymph node and highlights the possibility that preoperative SUV_LN_ could be a promising prognostic marker before radical surgery. More intensive attention should be paid to high SUV_LN_ group patients. Although the clinical benefit of the intensive surveillance strategy was not evaluated, earlier detection of recurrence might influence decision-making for treatment and may affect prognosis. This strategy should be confirmed in further prospective studies.

This study has several limitations. First, our study was in part limited by its retrospective design at a single institution with a small sample size. Further large prospective studies in different institutions are needed to validate the prognostic value of SUV_LN_ in patients with CRC. Nevertheless, our study is noteworthy because it is the first study to show the importance and prognostic value of preoperative SUV_LN_ in patients with CRC. Second, the area under the curve in the ROC analysis was 0.683, and the specificity using the cut-off of SUV_LN_ was only 59.2%. Although the sensitivity was 84.2%, low specificity at the SUV_LN_ cut-off value may be a problem in clinical practice. Third, we did not perform overall survival analysis because there were only six patients who were died during follow-up. Future large prospective studies are needed to investigate the relationship between SUV_LN_ and overall survival in CRC patients.

## 5. Conclusion

In conclusion, we provide for the first time the importance of metabolic activity of lymph nodes, showing that preoperative SUV_LN_ was an intuitive and simple method, significantly associated with recurrence in patients with CRC. Therefore, preoperative assessment of SUV_LN_ may be a promising prognostic marker to identify patients with a high risk of recurrence of CRC.

## Figures and Tables

**Table 1:** Characteristics of 90 patients with CRR who underwent PET/CT before radical operation.

Characteristics	Patients	%
*Age, median (range)*	66 (43–87)	
*Sex*		
Male	56	62.2
Female	34	37.8
*Tumour location*		
Colon	44	48.9
Rectum	46	51.1
*Mean tumour size, cm (range)*	4.67 (1.2–12.0)	
*T stage*		
T1/2	17	18.9
T3	47	52.2
T4	26	28.9
*Lymph node metastasis*		
No	63	70
Yes	27	30
*Lymphovascular invasion*		
No	78	86.7
Yes	12	13.3
*Tumour grade*		
Well/moderate	71	78.9
Poor	19	21.1
*Adjuvant treatment*		
No	31	34.4
Yes	59	65.6
*SUV*_*Tumor*_ *, median (range)*	20.01 (6.3–55.4)	
*SUV*_*LN*_ *, median (range)*	2.51 (0.4–16.9)	
*Recurrence*		
No	71	78.9
Yes	19	21.1

**Table 2:** Patient characteristics according to cancer recurrence.

Characteristics	Total (*n*=90)	No recurrence (*n*=71)	Recurrence (*n*=19)	*P*
*Age*		66.41 ± 10.27	65.05 ± 11.34	0.618
*Sex*				
Male	56	46	10	0.332
Female	34	25	9	
*Tumour location*				
Colon	44	32	12	0.161
Rectum	46	39	7	
*Tumour size*		4.57 ± 1.92	5.04 ± 2.56	0.382
*T stage*				
T1/2	17	16	1	0.023
T3	47	39	8	
T4	26	14	12	
*Lymph node metastasis*				
No	63	51	12	0.464
Yes	27	20	7	
*Lymphovascular invasion*				
No	78	64	14	0.120
Yes	12	7	5	
*Tumour grade*				
Well/moderate	71	61	10	0.004
Poor	19	10	9	
*Adjuvant treatment*				
No	31	27	4	0.167
Yes	59	44	15	
*SUV*_*Tumor*_		20.52 ± 10.77	18.11 ± 9.75	0.409
*SUV*_*LN*_		2.22 ± 0.32	3.58 ± 0.87	0.015

**Table 3:** The relationship between SUV_LN_ and site of recurrence.

Recurrence site	Number of patients (%)	SUV_LN_, median (range)	*P*
Distant metastasis	9	3.42 (0.7–10.2)	0.841
Peritoneal recurrence	4	3.0 (2.0–4.0)	
Locoregional recurrence	6	3.59 (0.7–15.2)	

**Table 4:** Regression analyses of prognostic factors for recurrence-free survival in patients with CRC.

Variable	Test for recurrence-free survival	Univariate analysis			Multivariate analysis		
		Hazard ratio	95% CI	*P*	Hazard ratio	95% CI	*P*
Age	>60 versus ≤60	0.657	0.262–1.647	0.371			
Sex	Male versus female	0.463	0.187–1.149	0.097			
Tumour location	Rectum versus colon	0.448	0.176–1.142	0.093			
Tumour size	>4.25 versus ≤4.25	2.336	0.885–6.172	0.087			
T Stage	3/4 versus 1/2	2.018	0.987–4.123	0.054			
Lymph node metastasis	Yes versus No	1.182	0.463–3.021	0.727			
Lymphovascular invasion	Yes versus No	2.396	0.842–6.815	0.101			
Tumour grade	Poor versus well/Moderate	3.143	1.272–7.767	0.013	3.837	1.501–9.81	0.005
Adjuvant treatment	Yes versus No	1.803	0.596–5.449	0.296			
SUV_Tumor_	>10.65 versus ≤10.65	1.613	0.601–4.334	0.343			
SUV_LN_	>1.15 versus ≤1.15	4.538	1.317–15.642	0.017	10.107	2.832–36.064	<0.0001

**Table 5:** Characteristics of patients categorized according to SUV_LN_.

Characteristics	Total (*n*=90)	SUV_LN_		*P*
		≤1.15	>1.15	
*Age*		67.33 ± 10.21	64.68 ± 10.68	0.234
*Sex*				
Male	56	28	28	0.277
Female	34	21	13	
*Tumour location*				
Colon	44	21	23	0.211
Rectum	46	28	18	
*Tumour size*		4.27 ± 1.87	5.14 ± 2.20	0.045
*T stage*				
T1/2	17	12	5	0.307
T3	47	23	24	
T4	26	14	12	
*Lymph node metastasis*				
No	63	39	24	0.03
Yes	27	10	17	
*Lymphovascular invasion*				
No	78	43	35	0.740
Yes	12	6	6	
*Tumour grade*				
Well/Moderate	71	40	31	0.486
Poor	19	9	19	
*Adjuvant treatment*				
No	31	19	12	0.345
Yes	59	30	29	
*SUV*_*Tumor*_		20.09 ± 10.40	19.91 ± 10.87	0.935
*Recurrence*				
No	71	46	25	<0.0001
Yes	19	3	16	

## Data Availability

The data used to support the findings of this study are available from the corresponding author upon request.
